# Screws

**Published:** 1874-03

**Authors:** 


### Editorial.

### SCREWS.

Almost every dentist has heard of "Mack's Screws." and many have used them. Some have the impression that he devised and first used screws for filling teeth. This is not correct; Dr. Dwinelle, of New York, described their use more than twenty years ago. We have used them occasionally for more than twenty years, and others have used them for many years. Mr. Mack is the only one who has had them manufactured, together with the appliances for their use, and placed in the market. For this he is entitled to credit, but not for the conception.

Notwithstanding the screw, as an aid in filling teeth, was described so long ago, and has for the last three years been advertised and in the market, still comparatively few have attempted to use it. Though it is in many cases invaluable, some operations are impracticable without it.

In capping the ends of teeth, or building upon a surface where there is no cavity and it is difficult or impracticable to form one, they render it possible, and indeed oftentimes quite easy, to make a very valuable and permanent operation. They may also be used very efficiently in large cavities where the retention of a filling depends wholly upon retaining pits. In such cases the screws afford a more secure anchorage or attachment than ordinary retaining pits or grooves. There is no liability of breaking the screws; and when two or more of them are set in place, even though it be upon a plain surface of a tooth at different angles, firmly screwed in,—there is no necessity of preceding them with a screw tap,—and gold welded between and around them, making firm their relative position, it is impossible to remove them without breaking the tooth, the divergence and thread of the screws making absolute security.

Some have made them with a head upon the outer end. This is not necessary, and especially so if the screw is the full length.

Some prefer to have the wire in length, with the screw thread upon it, and upon introducing seize it with a pin vice and introduce it to its position, then with the snips cut it off at the desired length.

Dr. Osmond, of this city, prepares screws in the same manner, except that he splits the end of the pin, and, when the larger part of the filling is introduced, bends down each half upon the filling, and in this way effecting greater security, as it is claimed.

We can hardly conceive of a case in which equal efficiency could not be secured by the proper use of Mack's Screws, or, indeed, any other screws. In the hands of the inventor there may be some points that others do not see or appreciate. Such is the case oftentimes. There is, with reference to many new things upon their introduction, a barrenness of accessory apparatus or appliances. Such is not the case, however, with Mack's Screws; for, instead of one screw-driver and two drills, all of which ought to be furnished for one dollar, there are nine instruments, sold at $12 to $18. In the outstart, in an enthusiastic haste, one might be pardoned for error in this direction; but, after having had time for reflection and correction, it is an imposition that an unnecessary expenditure of $10 to $16 shall be made by every one that uses Mack's Screws.

That's putting the screws to the profession pretty strong; but they are used to it and don't wince very much.

*Use the Screws.*

